# Prenatal diagnosis of rearrangements in the fetal 22q11.2 region

**DOI:** 10.1186/s13039-020-00498-y

**Published:** 2020-07-08

**Authors:** Suping Li, Yuxia Jin, Jing Yang, Li Yang, Ping Tang, Chiyan Zhou, Liping Wu, Jinhua Dong, Jie Chen, Huaxiang Shen

**Affiliations:** Department of Prenatal Diagnosis Center, Jiaxing University Affiliated Women and Children Hospital, East Zhong huan Road, 2468#, Jiaxing, 314050 Zhejiang China

**Keywords:** 22q11.2 deletion syndrome, 22q11.2 duplication syndrome, Prenatal diagnosis, Single-nucleotide polymorphism-array analysis, Chromosome analysis

## Abstract

**Background:**

22q11.2 deletion syndrome (22q11.2DS) and 22q11.2 duplication syndrome (22q11.2DupS) are the most common copy number variations in humans. The clinical phenotypes of these two syndromes are variable, and there are no large sample data on the prenatal detection rate for these two syndromes in the Chinese population.

**Results:**

We recruited 411 pregnant women who showed either abnormal prenatal ultrasound findings or positive prenatal BoBs™ results or who had given birth to a child with chromosomal abnormalities. SNP-array analysis and interphase FISH analysis identified five fetuses with 22q11.2 copy number variants (CNVs), three of which were 22q11.2 deletion syndrome (22q11.2DS) (3/411) and two of which were 22q11.2 duplication syndrome (22q11.2DupS). In all 5 cases of diagnosed 22q11.2 abnormalities, inheritance could not be identified because the parents did not undergo further testing.

**Conclusion:**

Our case reports provide a detection rate of 22q11.2 CNVs for fetuses with prenatal diagnostic indications, and early diagnosis of these two syndromes was essential for prenatal intervention in these cases. SNP-array technology is an effective tool in the prenatal diagnosis of 22q11.2 CNVs. The prenatal diagnosis of these two syndromes is helpful for early intervention, which is of great clinical significance.

## Background

22q11.2 deletion syndrome (22q11.2DS) is one of the most common and best-characterized recurrent chromosomal deletion syndromes [1], with an incidence of approximately 1 in 1000 fetuses [2] and 1 in 4000 live births [3]. Most chromosome 22 genomic abnormalities are due to low copy repeat sequence (LCR)-mediated nonhomologous dislocations and uneven intrachromosomal or interchromosomal recombination during meiosis. The 22q11.2 region is rich in LCRs, making this region a high incidence area of chromosome 22 abnormalities as a result of both intrachromosomal or interchromosomal nonallelic homologous recombination events [4]. Most patients with 22q11.2DS have congenital heart disease, immune deficiency, transient neonatal hypocalcemia, velopharyngeal insufficiency, a distinctive facial appearance, variable cognitive delays, behavioral anomalies, and psychiatric illness [1, 2].

A duplication interchromosomal event in the same region of chromosomal 22q11.2 causes a syndrome referred to as 22q11.2DupS [5, 6]. It is thought that the morbidity of 22q11.2DupS is half that of 22q11.2DS. This syndrome is characterized by a highly variable clinical phenotype, which ranges from apparently normal or slightly dysmorphic features with moderate learning disabilities to severe malformations with profound intellectual disability [5, 7, 8]. The birth of children with 22q11.2DS or 22q11.2DupS causes extreme emotional and financial burdens on society and the family. However, very few studies have evaluated the performance of prenatal diagnosis and assessment in managing these diseases.

In the present study, we retrospectively analyzed prenatal clinical features and performed single-nucleotide polymorphism chromosome microarray technology (SNP-array analysis)-based diagnostic detection in 411 pregnant women recruited during the past 4 years. These results may be useful for pregnant women and their families regarding the prognosis of the fetus before delivery.

## Materials and methods

### Patients

The present study was carried out according to the principles of the Declaration of Helsinki and approved by Jiaxing Maternity and Child Health Care Hospital Affiliated with Jiaxing University’s Ethics Committee. Written informed consent was obtained for all human subjects participating in this case series study. From January 2015 to December 2018, a total of 411 pregnant women were recruited, among whom 275 presented with structural abnormalities in the fetus during ultrasound scanning; the remaining 136 were identified to be at high risk by Down’s serological screening and were subsequently confirmed to have chromosomal abnormalities by amniotic fluid karyotype analysis or prenatal BoBs test.

### Amniotic fluid karyotype analysis

Amniotic fluid (20 mL) was collected by amniocentesis under ultrasonic guidance and then centrifuged at 800×*g*/min for 10 min. After removing the supernatant, the remaining 1.5 mL of liquid was mixed and inoculated in two 3.5-mL culture bottles containing AmnioMAX™ II Complete culture medium (BIOAMF™-2, Biological Industries, Inc.). The culture medium was changed 6–7 days after inoculation. The cells were collected, and sections were prepared for Giemsa banding when more than 10 cell colonies were present. Images were scanned and collected by using a Leica Biosystems GSL-120 and Lecia CytoVisin analysis system (California Richmond, USA). We analyzed 5 mitotic figures and 30 counts for each sample.

### Prenatal BACs on BEADS™ (BoBs™) technology

Prenatal BoBs™ (Luminex 200, Technology Boulevard Austin, Texas, USA) is a rapid, microsphere-based suspension array technology used to discover common aneuploidies (13, 18, and 21) and nine frequent microdeletion/microduplication syndromes, including Williams-Beuren, DiGeorge I and II, Prader-Willi, Angelman, Cri du Chat, Smith-Magenis, Langer-Giedion, Miller-Dieker and Wolf-Hirschhorn.

DNA was extracted from amniotic fluid cells and detected with Prenatal BoBs™ technology. Positive results were supported by SNP-array analysis.

### SNP-array analysis

High-resolution, genome-wide SNP-array analysis was performed using the Affymetrix CytoScan 750 K arrays technology platform (Santa Clara, CA, USA) according to the manufacturer’s protocols. The results were analyzed with ChAS v3.0 software (Thermo Fisher Scientific, Waltham, MA, USA).

### Fluorescence in situ hybridization (FISH)

Fluorescence in situ hybridization analysis (using the Di George syndrome-specific flanking N25 region probe coupled with an LSI ARSA control probe that maps to the telomeric end of 22q13) was performed according to the manufacturer’s instructions (Abbott Laoratories, USA) for at least 30 interphase nuclei. A FISH result for interphase nuclei with three separate red fluorescent signals indicated a duplication event.

## Results

Among the 411 pregnant women who showed either abnormal prenatal ultrasound findings or positive prenatal BoBs™ results or who had given birth to a child with chromosomal abnormalities, SNP-array analysis identified five fetuses with 22q11.2 copy number variants (CNVs), three of which were 22q11.2DS and two of which were 22q11.2DupS (Table 1). Except for the P5 fetus with a duplication of 2.8 Mb, the copy number variations of the other four fetuses were all approximately 3.1 Mb. The prenatal positive detection rate of 22q11.2 microdeletion was 7.30 in 1000 fetuses (3/411), and the positive detection rate of 22q11.2 microduplication was 4.87 in 1000 fetuses (2/411). These figures reflect the incidence of 22q11.2 CNV high-risk populations in China.

**Table 1:** Pregnancy details and clinical features of the patients

Patient	Age	G/P/A/L	Gestational week	T21	T18	NTD Risk	NT (mm)	Syndrome	CNVs Size	Clinical indication	Outcome
P1	25	0–0–0-0	23	1/2300	1/36400	Negative	0.9 mm	22q11.2DS	3.1 Mb	Fetal heart malformation, separation of left kidney collecting system	Termination of pregnancy
P2	25	0–0–0-0	23	1/6824	1/100000	Negative	5.8 mm	22q11.2DS	3.1 Mb	Fetal neck water cyst	Termination of pregnancy
P3	30	1–0–0-1	22	1/3777	1/100000	Negative	Not done	22q11.2DS	3.1 Mb	Fetal ventricular septal defect slight tricuspid regurgitation	Continue pregnancy
P4	30	1–0–0-1	21	1/53	1/22509	Negative	1.2 mm	22q11.2DupS	3.1 Mb	BoBs result:22q11.2 region duplication	A 2 years old healthy child
P5	31	0–0–0-0	22	1/20	1/25507	Negative	1.7 mm	22q11.2DupS	2.8 Mb	BoBs result:22q11.2 region duplication	Termination of pregnancy

**Fig. 1:**
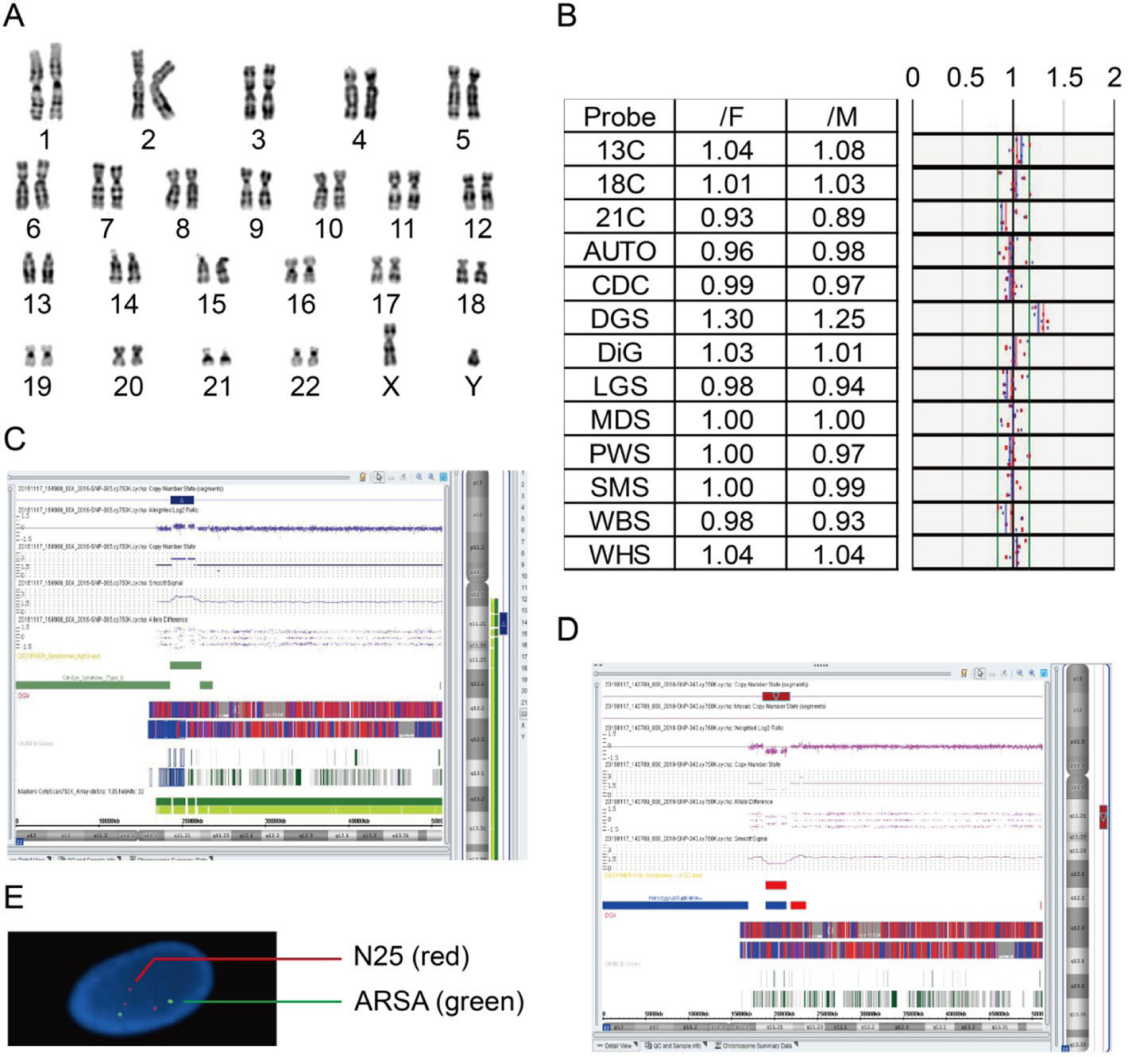
Karyotype analysis, BoBs™ analysis, SNP-array analysis, and FISH analysis. **a** Karyotype of P3, normal karyotype. **b** BoBs™ analysis of P5. The arrow indicates DiGeorge syndrome region amplification. **c** SNP-array analysis of P5. The thick blue line indicates duplication of the 22q11.2 region. **d** SNP-array analysis of P3. The thick red line indicates deletion of the 22q11.2 region. **e** FISH analysis of P5. N25 (red) indicates duplication of the DiGeorge region

G-banded chromosome analysis of prenatal amniotic fluid cells revealed normal karyotypes in all 5 confirmed fetuses (Fig. 1a). Prenatal BoBs™ technology only indicated an increased signal in two fetuses in the 22q11.2 region (Fig. 1b) but was unable to determine the size and genes contained in the region. The SNP-array analysis technique was able to identify the size of amplification and the genes in this region (Fig. 1c). The three fetuses with 22q11.2DS and ultrasound abnormalities were directly diagnosed by SNP-array analysis (Fig. 1d). Interphase FISH analysis identified one of the 22q11.2 microduplications (P5) by using the Di George syndrome-specific flanking N25/ARSA probe (Fig. 1e). In all 5 fetuses with the diagnosed 22q11.2 abnormalities, inheritance could not be identified because the parents did not undergo further testing.

**Fig. 2:**
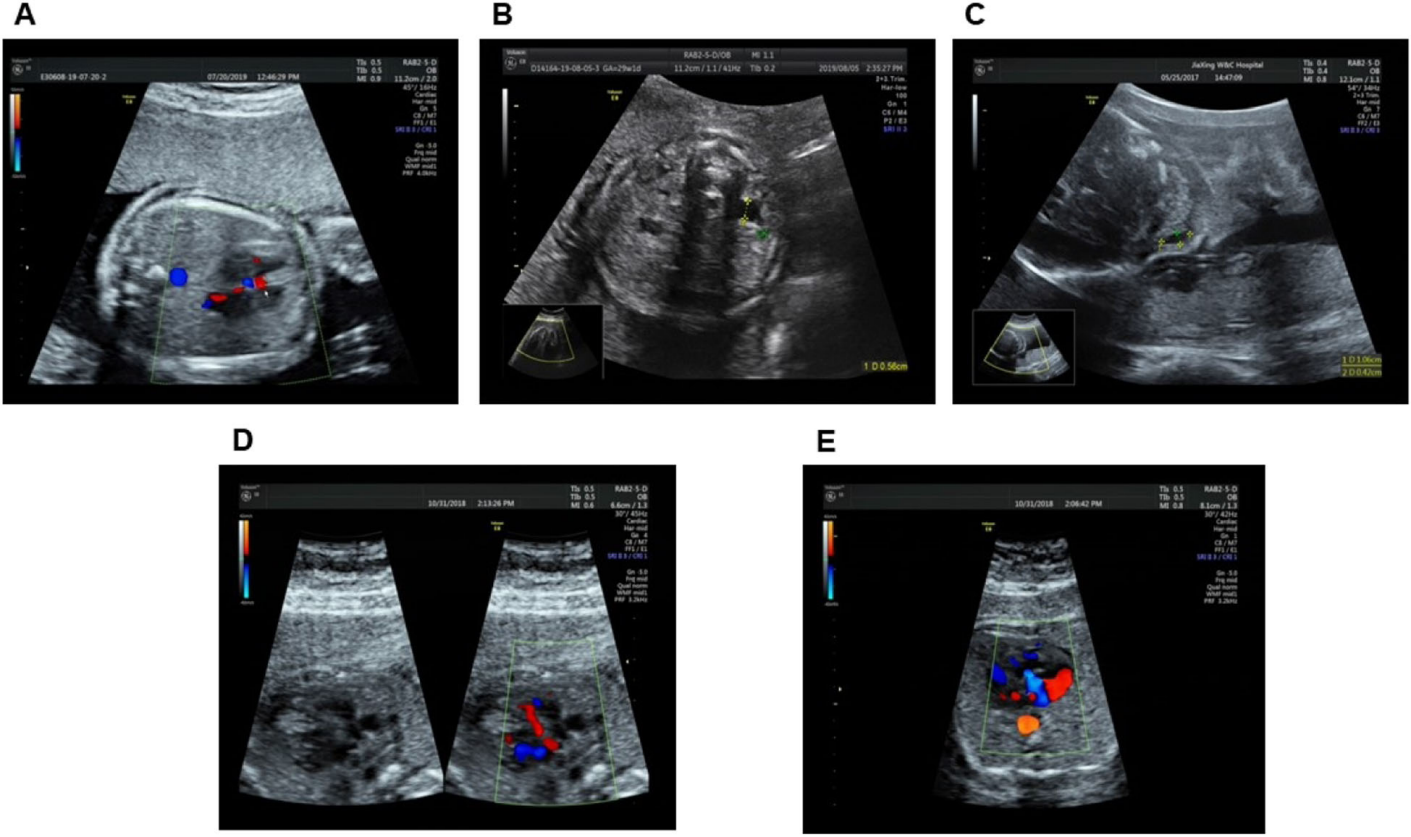
Prenatal ultrasonographic findings of the fetus. **a** P1 fetus at 23 weeks gestational age, showing ultrasonographic findings of tetralogy of Fallot. **b** P1 fetus, showing ultrasonographic findings of left kidney collection system separation. **c** P2 fetus, showing ultrasonographic findings of a water cyst in the neck. **d** P3 fetus, showing ultrasonic findings of tricuspid regurgitation. **e** P3 fetus, showing a perimembranous ventricular septal defect

Prenatal fetal ultrasound scanning of P1 at 23 weeks gestational age showed tetralogy of Fallot (Fig. 2a) and left kidney collection system separation (Fig. 2b). The double top diameter, head circumference, abdominal circumference, and femur length were all within the normal range, and the spine continuity was complete. Prenatal ultrasound of the P2 fetus suggested a water cyst in the neck (Fig. 2c), and other growth indications were within the normal range. The parents of P1 and P2 chose to terminate the pregnancy. P5 at 22 weeks gestational age did not reveal any abnormal prenatal ultrasound findings. However, the parents chose to terminate the pregnancy because of fear of abnormalities in the neurologic development after birth after receiving the genetic diagnosis of 22q11.2DupS.

Echocardiography of the P3 fetus revealed a 0.28 cm perimembranous ventricular septal defect (Fig. 2d) and tricuspid regurgitation (Fig. 2e). The ascending aorta was slightly distorted; other growth indicators were within the normal range. After genetic counseling, the pregnant woman and her husband choose to continue the pregnancy, and the baby had not yet been delivered at the time of writing. Prenatal ultrasound of the P4 fetus did not reveal any anomalies, so the pregnant woman chose to continue the pregnancy and have a normal birth. The child is now a 2-year-old girl, and physical and mental examinations did not reveal any abnormalities (height 86 cm, weight 12 kg, head circumference 48 cm, heart and lung without murmur, liver and spleen without swelling, no skeletal abnormalities, normal speech and behavior development).

## Discussion

22q11.2DS, otherwise known as DiGeorge syndrome or velo-cardio-facial syndrome, is a well-described genetic syndrome with clinical presentations including congenital heart defects and other malformations. Previous studies did not detect the 22q11.2 deletion in controls, confirming the pathogenic nature of this CNV [9, 10]. However, 22q11.2DS syndrome is an underdiagnosed disease in the general population, especially in developing countries. In China, patients with conotruncal defects or 22q11.2DS-specific facial features are underrecognized because it is difficult to recognize the disease solely based on clinical features without knowing the molecular diagnosis [11, 12]. In our three cases of 22q11.2DS, two fetuses had congenital heart malformations and one had a water cyst on prenatal ultrasound findings. This indicates that fetuses with such ultrasound abnormalities may undergo karyotype analysis and SNP-array analysis to confirm genetic diagnosis.

In the present study, we retrospectively analyzed the clinical features of fetuses with 22q11.2DS and 22q11.2DupS analyzed over the past 4 years by SNP-array analysis to provide a clinical basis for prenatal diagnosis of these two common syndromes. Despite the small number of cases, this is the first time that Chinese data for the diagnosis of 22q11.2 CNV by the SNP-array technique have been obtained by amniocentesis. Our detection rate of 22q11.2DS (1:137) is similar to that reported by Kaminsky et al. (1/169), but 22q11.2DupS shows a lower detection rate (1:205.5), probably because of its milder phenotype [9, 13]. However, the overall positive detection rates in our center for the two syndromes are significantly higher than those reported previously [1, 3, 8], probably due to our patient selection criteria. The diagnosis would be more convincing if we could obtain the CNV data of the 5 couples with 22q11.2 region-problem pregnancy. However, such information was not available due to refusal of further inspections from the parents at the time of publication.

Prenatal BoBs™ and SNP-array analysis have become popular diagnostic technologies for common aneuploidies and other microdeletion or microduplication syndromes in the past several years in China [14]. Prenatal BoBs™ is a bead-based multiplex technology that detects chromosome 13,18, 21, and X/Y aneuploidies and nine frequent microdeletion syndromes [15, 16]. However, whole-genome SNP-array analysis has demonstrated higher resolution and more accurate CNV detection than BoBs™ technology [9]. In addition, SNP-array analysis can detect many other pathogenic/likely pathogenic microdeletions/duplications in addition to aneuploidy/partial aneuploidy, triploidy, UPD, and other disorders [17]. FISH detection had been the gold standard for identifying microdeletions and microduplications. However, owing to the rapid development of new technologies, it is currently used as a verification method for known variations. Traditional karyotype analysis cannot distinguish copy number variations < 5 Mb. Although FISH detection is accurate, it may miss a small fragment or an atypical variation. BoB detection can only provide an indication of the amplification or deletion of the probe signal position. SNP-array technology accurately detects the position, size and genetic content of copy number variations. Therefore, SNP-array technology shows a unique advantage in detecting unknown copy number variations, including 22q11.2 CNVs.

22q11.2DupS is associated with highly variable clinical features, ranging from completely normal to slightly abnormal features with milder learning disabilities to severe intellectual disability [5, 18]. Therefore, very few cases have been reported, and the absence of obvious clinical features makes diagnosis difficult [19]. Similarly, no ultrasound abnormalities were observed in the two cases of 22q11.2DupS in this study. The parents of one fetus chose to give birth to the child; the child is now 2 years old and healthy. The other pregnant woman chose to terminate the pregnancy because of concerns regarding their child’s potential postnatal mental problems. These results and those from the literature [8] suggest that 22q11.2DupS does not have an accurate clinical phenotype. The extent of correlation between the phenotype and CNV depends on many factors, including previous evidence of pathogenic CNVs in the same region, type of CNV (duplication or deletion), gene content, inheritance pattern, and frequency in healthy populations [9, 19]. Our case reports have provided useful information for subsequent research and genetic counseling. The collection of clinical data from larger-scale studies of 22q11.2DupS is necessary for this type of analysis to provide evidence for prenatal diagnosis and genetic counseling.

## Conclusion

Our case reports provide a detection rate of 22q11.2 CNVs for fetuses with prenatal diagnosis indications and further indicate that SNP-array technology is an effective tool in the prenatal diagnosis of 22q11.2 CNVs. The prenatal diagnosis of these two syndromes is helpful for early intervention, which is of great clinical significance.

## Data Availability

The data used or analyzed during the current study are avaible from the corresonding author on reasonable request.
